# A Gellan Gum, Polyethylene Glycol, Hydroxyapatite Composite Scaffold with the Addition of Ginseng Derived Compound K with Possible Applications in Bone Regeneration

**DOI:** 10.3390/gels10040257

**Published:** 2024-04-10

**Authors:** Muthukumar Thangavelu, Pil-Yun Kim, Hunhwi Cho, Jeong-Eun Song, Sunjae Park, Alessio Bucciarelli, Gilson Khang

**Affiliations:** 1 Linkocare Life Sciences AB, Mjärdevi Science Park, 583 30 Linköping, Sweden; 2 Department of Bionanotechnology and Bio-Convergence Engineering, Jeonbuk National University, 567 Baekje-daero, Deokjin-gu, Jeonju-si 54896, Jeonbuk, Republic of Korea; kpy@jbnu.ac.kr (P.-Y.K.); hhcho@jbnu.ac.kr (H.C.); songje@jbnu.ac.kr (J.-E.S.); 3 Department of Polymer Nano Science & Technology and Polymer Materials Fusion Research Center, Jeonbuk National University, 567 Baekje-daero, Deokjin-gu, Jeonju-si 54896, Jeonbuk, Republic of Korea; sunjaepark@jbnu.ac.kr; 4 Laboratorio RAMSES, IRCCS Istituto Ortopedico Rizzoli, Via di Barbiano 1/10, 40136 Bologna, Italy

**Keywords:** scaffold, bone tissue engineering, gellan gum, hydroxyapatite, ginseng compound K

## Abstract

Engineered bone scaffolds should mimic the natural material to promote cell adhesion and regeneration. For this reason, natural biopolymers are becoming a gold standard in scaffold production. In this study, we proposed a hybrid scaffold produced using gellan gum, hydroxyapatite, and Poly (ethylene glycol) within the addition of the ginseng compound K (CK) as a candidate for bone regeneration. The fabricated scaffold was physiochemically characterized. The morphology studied by scanning electron microscopy (SEM) and image analysis revealed a pore distribution suitable for cells growth. The addition of CK further improved the biological activity of the hybrid scaffold as demonstrated by the MTT assay. The addition of CK influenced the scaffold morphology, decreasing the mean pore diameter. These findings can potentially help the development of a new generation of hybrid scaffolds to best mimic the natural tissue.

## 1. Introduction

Bone is a complex hierarchical tissue with a high regenerative capacity. Approximately 15% of our body weight is made up of bone, consisting of minerals (primarily hydroxyapatite, accounting for 60%), organic components (mostly type-I collagen, making up 30%), and water (10%) [1,2,3]. This composition directly reflects the three main functions of this tissue: providing mechanical stability by responding to the external mechanical stimuli, protecting organs by containing them, and serving as a mineral reserve [2]. Due to its self-healing capabilities, bones promote the spontaneous repair of small fractures without scar formation by activating a complex and dynamic repair process divided into three distinct phases: inflammation, bone formation/renewal, and bone remodeling [4]. In the first phase, the activation of the inflammatory cascade leads to the formation of the hematoma and the soft granulation tissue [5]. During the bone formation phase, a heterogenous tissue, which initially consists of a soft and later a hard callus, forms the woven bone [6]. Finally, during the remodeling phase, ossification occurs, and the woven bone is substituted by lamellar bone tissue [7]. During this dynamic process, the tissue is continuously remodeled and exhibits different properties in each phase, which involves different cells and metabolites, angiogenesis, and most importantly, the increase in mechanical strength, changing from the soft granulation tissue to the final stiff remodeled bone [8,9].

Damage to bone tissue can be caused by trauma, diseases, injuries, and surgery [10]. In several of these cases, the severity of the damage does not allow autonomous bone regeneration, and the patient must be implanted with additional tissue to ensure complete recovery. For example, when bone resorption is no longer compensated for by bone formation, there is a progressive decline in bone mass, ultimately leading to osteoporosis, a major risk factor for fragility fractures [11]. For this purpose, various materials and surgical procedures have been investigated over the years. Autograft bone transplantation, which consists of implanting on the defective site bone harvested from a healthy site of the patient, is still considered the gold standard. Since the implanted tissue contains the patient’s own cells, the autograft bone supports osteoinduction, osteoconduction, and osteogenicity, thereby minimizing the risk of an immunological response [12]. This procedure is often replaced by the implantation of bone from a donor (allograft bone surgery) particularly when the recipient suffers from bone-related diseases (e.g., osteoporosis) that compromise the structural integrity of their own bone [13]. Both strategies have their own intrinsic limitations. Blood loss, inflammation, infection, and persistent pain are among the surgical risks associated with autograft bone transplantation, which can also cause severe donor site damage, morbidity, deformity, and scarring [14,15]. On the other hand, compared to autografts, allografts are more expensive and have a slower rate of osteointegration, a higher risk of resorption, and an infection [16,17].

Bone tissue engineering (BTE) is focused on developing engineered scaffold systems suitable for bone replacement with the aim of avoiding all the above problems. The core idea of BTE is the development of constructs that can mimic bone structures and thus guide cells to ensure the regeneration of the native bone tissue. An optimal scaffold should provide mechanical support for bone regeneration, provide an appropriate microenvironment for cell adhesion and proliferation, have sufficient porosity to allow the circulation of nutrients and the reorganization of cells, and degrade according to the formation of the new tissue [18,19,20].

The source of the material chosen to build the scaffold plays a key role in bone regeneration. In fact, the material must be biocompatible and potentially promote cell differentiation as well as ensure adequate mechanical strength, which largely depends on its properties. Both natural and synthetic polymers are commonly used to produce scaffolds [21]. Natural polymers have emerged as favorable candidates to produce biomimetic scaffolds for bone regeneration due to their superior biological properties [22,23,24,25,26,27], while synthetic polymers have higher mechanical properties [28,29]. However, synthetic polymers usually have inferior biocompatibility and lower bioactivity compared to their natural counterpart. In addition, the byproduct of the synthetic polymers’ degradation has been proved to cause aseptic inflammation when implanted in larger animals and humans [30]. For these reasons, synthetic polymers are often blended with natural polymers, which improve their biological properties [31].

Gellan gum (GG) is an anionic exocellular heteropolysaccharide produced by bacteria of the Sphingomonas group as a major component of their extracellular polymeric substance (EPS). GG has already been used to develop scaffolds for bone regeneration [32,33,34,35] in combination with other materials able to both improve mechanical properties and enhance biological responses. For bone application, GG has been loaded with several materials, including bioglasses [36], nano hydroxyapatite [35], and demineralized bone matrix [37], among others. These materials both simulate the mineral components of natural tissue and increase stiffness [35,36,37]. In particular, the use of hydroxyapatite (HA, the main inorganic component of bone) has been proved to support osteoconduction and osteointegration during bone repair [38,39]. Poly (ethylene glycol) (PEG) is a biocompatible, FDA-approved, synthetic polymer that is commonly used in tissue engineering and proved to be beneficial in in vivo bone regeneration [40,41]. PEG can also be blended with natural polymers to improve their mechanical stability, thus forming composite scaffolds [40,41].

A common strategy to improve the biological response and achieve faster bone regeneration is the inclusion of bioactive molecules in the scaffold. Compound K (CK) is a ginsenoside metabolite extracted from ginseng, which is a plant commonly used as a supplement in several countries throughout the world [42]. The metabolism of ginsenosides Rb1, Rb2, and Rc by intestinal bacteria in vivo results in the production of CK [43]. Human intestinal bacteria metabolize protopanaxadiol type ginsenosides through a metabolic pathway consisting of Rb1, Rb2, or Rc, resulting in the formation of Rd, F2, and CK [44]. CK was also proven to significantly increase the mRNA expression of several genes (Wnt10b, Wnt11, Lrp5, and β-catenin) regulating Wnt/β-catenin signaling [45], which is directly involved in bone formation and osteoblast differentiation [46]. Based on this assumption, CK has been proven to be beneficial in the case of bone fractures by promoting osteogenesis and angiogenesis [47], effective in inhibiting RANKL-induced osteoclastogenesis [48]. However, CK has rarely been used in the development of bone scaffold systems.

In this study, we developed starting from a crosslinked hydrogel a porous composite hybrid scaffold based on GG, PEG, and HA with potential application in bone tissue engineering, investigating the effect of the addition, namely compound K (CK). We characterized the scaffold morphology (by secondary electron microscopy), mechanical stiffness (by compression), chemical structure (by Fourier infrared and X-ray spectroscopy), thermal behavior (by differential scanning calorimetry and thermogravimetric analysis), swelling and degradation. An initial assessment of the cell vitality was performed on adipose-derived mesenchymal stem cells (using MTT assay).

## 2. Results and Discussion

In this work, we developed and characterized a composite scaffold with a possible application in bone tissue regeneration. Gellan gum within PEG provided a support matrix to integrate the other components. The first material was chosen because of the demonstrated ability to form after freeze-drying a suitable porous microstructure to allow osteoconduction [49], while the second was selected to stabilize the matrix for longer times and to give to the structure a certain degree of tensile strength which is necessary to support other mechanical stresses than compression [50]. Hydroxyapatite was used to improve the mechanical performances in compression and because, being the major mineral component of bone, it favorably impacts the biological outcome [35]. CK was rarely used in bone scaffolding, but it has been proven to be beneficial in the case of fractures [47], which makes it a good candidate to be used in bone tissue engineering. We characterized a series of scaffold with increasing concentrations of HA, and to the one with the higher concentration, we added CK. The scaffold was nominated by separating the different components with colons. For instance, GG:PEG:HA20%:CK indicates the addition of 20% in the weight of HA to the GG:PEG scaffold and the addition of CK which was always added to the final concentration of 10 mM.

### 2.1. Morphological Analysis

**Figure 1:**
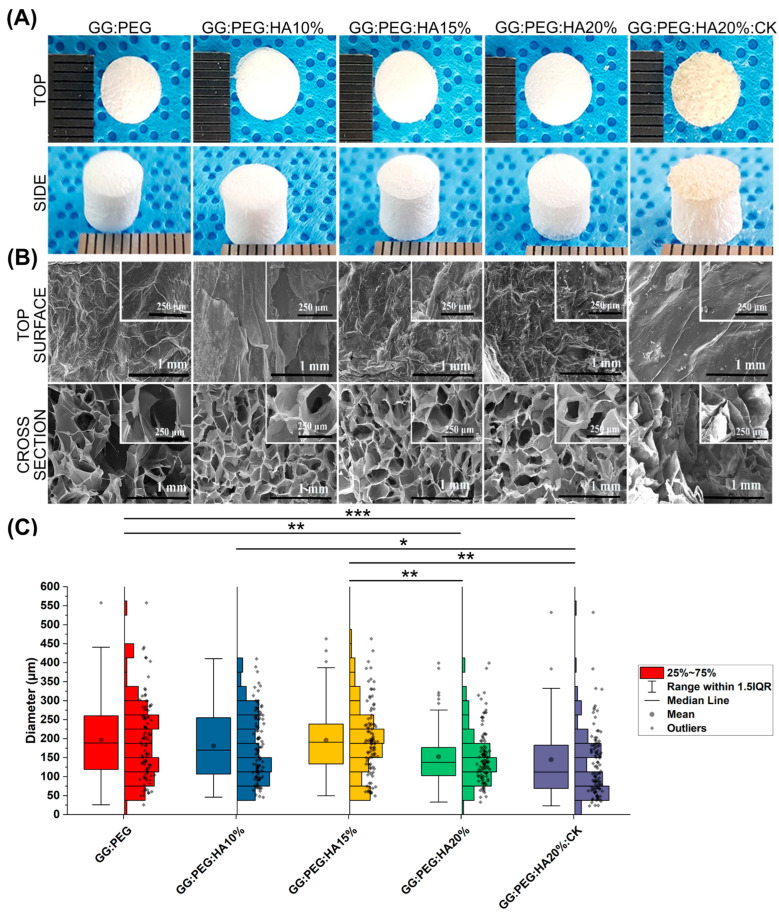
(**A**) The prepared scaffold from the top and side view after lyophilization. (**B**) SEM images of the surface and the cross section from the cross-sectional images the pore diameter distribution has been derived. (**C**) Boxplot within the data points and the histograms of each distribution. Significant differences have been found. In particular, the last group (with the addition of CK) has a significantly lower mean diameter compared with all the other groups. The significance levels of the ANOVA test were assigned as follows: *p* ≤ 0.05 (*), *p* ≤ 0.01 (**), *p* ≤ 0.001 (***). A difference was considered statistically significant if *p* ≤ 0.05.

Figure 1A illustrates the macroscopic images of the prepared scaffolds. Upon initial observation, all the scaffolds appeared to be identical except for the one with CK added. The GG:PEG:HA20%:CK scaffold exhibited a slightly darker color and distinct texture in comparison to the other scaffolds. This result was further confirmed by SEM. In particular, when examining the cross-section images shown in Figure 1B, it can be observed that the scaffold containing CK exhibited a distinct morphology characterized by a more lamellar structure, in contrast to the other scaffolds, which led to the formation of well-defined shaped pores. The morphology of all scaffolds, excluding the one with CK, displayed comparable characteristics. These characteristics include elliptical or spherical interconnected pores that are separated by thin walls measuring 1–2 µm in thickness. The SEM of the surfaces, especially in high magnification, revealed the presence of the HA particles entrapped in the scaffold. The diameter distributions, calculated from the cross-sectional images, are reported in the box graph in Figure 1C, and the statistical measures are summarized in Table 1. Interestingly, the addition of HA particles resulted in a slightly decreased pore diameter (both in terms of mean and median). However, it was only after adding 20% in the weight of HA that this difference became significant when compared to the GG:PEG scaffold. The presence of HA particles led to significant differences in the scaffold. The diameter of GG:PEG:HA10% and GG:PEG:HA15% was found to be significantly greater than that of the scaffold with 20% of added HA particles (both with and without CK). No statistical difference was found on the mean diameter among scaffolds with 15% and 10% HA particles added. In terms of the mean pore diameter, the presence of CK did not cause a significant alteration when compared to the scaffold without CK (GG:PEG:HA20%:CK). Both GG:PEG:HA20% and GG:PEG:HA20% exhibited similar pore sizes; however, they displayed distinct morphologies and distributions. Comparatively, the size distribution of GG:PEG:HA20%:CK was significantly skewed (Skewness = 2.63) and displayed a pronounced heavy tail (Kurtosis = 9.70) when contrasted with the raw scaffold (GG:PEG:HA20%).

**Figure 2:**
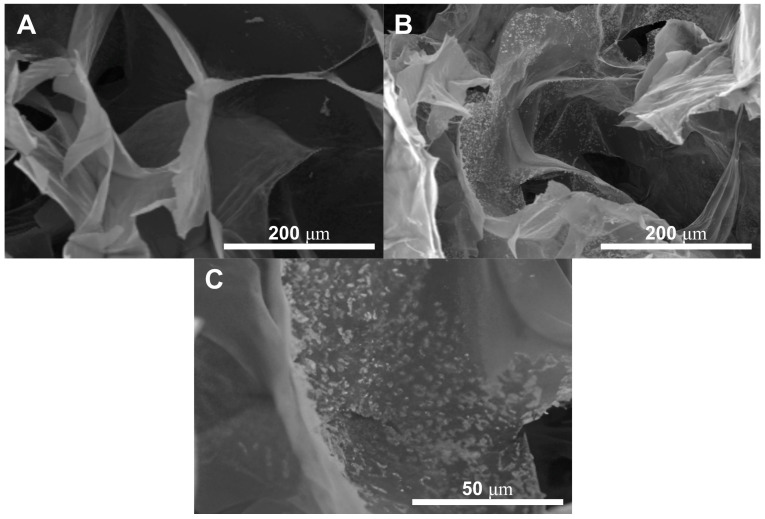
The presence of the HA nanoparticles has been confirmed by SEM through the comparison between (**A**) GG:PEG and (**B**) GG:PEG:HA20%:CK. The nanoparticles covered the scaffold surfaces. (**C**) Higher magnification showing the nanoparticles included in the scaffold surface. The particles appeared to be evenly distributed with the formation of several small agglomerates.

The presence of the HA nanoparticles was confirmed by SEM and are reported in Figure 2, particularly Figure 2C comparing the GG:PEG scaffold (Figure 2A) with the GG:PEG:HA20% scaffold (Figure 2B). The nanoparticles were evenly spread on the scaffold surface.

The ideal pore size for the regeneration of bone tissue is contingent upon the specific tissue being treated. A framework containing both macropores and micropores facilitates cell attachment, proliferation, and neovascularization. The minimum requirement for pore size, typically acknowledged, is 100 μm [51]. This is attributed to factors such as cell size, migration requirements, and nutrient transport. Nevertheless, it is recommended to have pore sizes greater than 300 μm, as this promotes enhanced formation of new bone and the development of capillaries [51]. The progression of osteogenesis has been observed to be influenced by pore size, owing to vascularization. Hypoxic conditions were favored by small pores and resulted in the formation of osteochondral tissue prior to osteogenesis. Conversely, large, well-vascularized pores promoted direct osteogenesis without preceding cartilage formation [51]. Our sponges having pores distributed in a large range of diameter (25–730 μm considering the minimum and maximum diameter detected) with a mean value in the 145–195 μm range results in all to be suitable as scaffolds for bone regeneration.

### 2.2. Mechanical Properties

**Figure 3:**
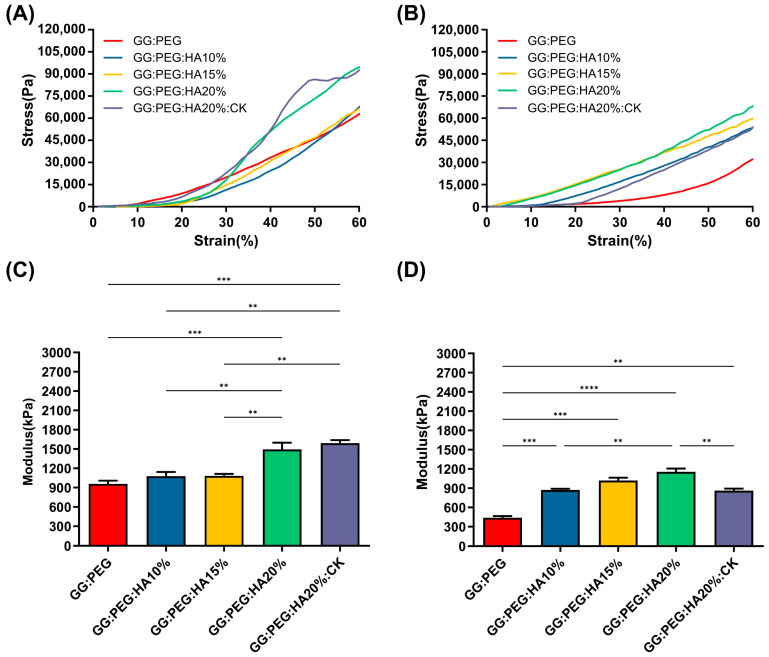
Stress–strain curve of (**A**) dry and (**B**) wet scaffolds, a clear reduction in the compressive modulus is visible. The trend of the compressive modulus is not immediately clear. (**C**) The compressive modulus for the dry sponges shows that both the addition of PEG and HA are significantly beneficial in terms of enhancement of the mechanical response. However, to see a significant effect, at least 20% of HA should be added to GG:PEG. The addition of CK does not affect the mechanical resistance in comparison with the bare scaffold. (**D**)The importance of the presence of the HA as filler became evident in wet conditions. In fact, the percentage of HA gave a significantly higher compressive modulus in comparison to the GG:PEG scaffold. Also, in this case, the addition of PEG was beneficial for the mechanical response of the scaffold. The inclusion of CK resulted in a decrease in modulus compared to the untreated scaffold, although it still remained higher than the modulus of the GG:PEG scaffold. The significance levels of the ANOVA test were assigned as follows: *p* ≤ 0.01 (**), *p* ≤ 0.001 (***), *p* ≤ 0.0001 (****). A difference was considered statistically significant if *p* ≤ 0.05.

The results of the mechanical test are shown in Figure 3. The mechanical curves of dry sponges (Figure 3A) and wet sponges (Figure 3B) clearly show the decreasing of the mechanical performances under wet conditions. The results also clearly show that a trend is present in both conditions within the increase in the amount of HA added. Both the addition of PEG and HA had a positive impact on the compressive modulus, reported in Figure 3C (dry condition) and Figure 3D (wet condition). The compressive modulus exhibited a significant decrease under wet conditions. Interestingly, the scaffolds with the higher loss in wet conditions compared to the dry condition were the GG (with a loss of 78%), followed by the GG:PEG (loss of 54%) and by the scaffold with the addition of CK (loss of 46%). In Table 2, we summarized the compression modulus, and all the scaffolds with GG, PEG, and HA in wet conditions had a compressive modulus higher than 500 kPa and, in general, a higher modulus than the GG:PEG scaffold. The presence of CK led to a small, non-significant increase in the compressive modulus compared to the bare scaffold (GG:PEG:HA20%) under dry conditions. However, under wet conditions, the CK scaffold showed greater losses in mechanical performance. The reason for this could be attributed to the difference in the pore structure of the CK scaffold. In fact, as previously reported, CK had an impact on the pore microstructure, resulting in a lamellar structure. It should be noticed that the impact of the pore geometry on the mechanical properties has been previously proven in the literature in simulated systems [52] and observed on scaffolding systems [53,54]. As expected, the increment in the mechanical properties in the wet condition was proportional to the amount of HA added, with the mineral components not susceptible of swelling.

The mechanical properties of our sponges are far from the ones found in a healthy bone tissue (130–200 MPa, compressive modulus of cortical bone [55,56]; 0.1–16 MPa, compressive modulus of trabecular bone [55,57]). However, in the early stages of bone formation after a fracture, the formed bone callus has mechanical properties extremely different from the healthy bone. The tissue in this stage is heterogenous with a compression modulus in the 0.5–500 MPa range [8]. Therefore, although the mechanical properties of the studied sponges did not correspond to those of a healthy natural bone, they exhibited mechanical characteristics consistent with previous research when considered as supportive aids during the early stages of fractures [6,7,8,58].

### 2.3. Chemical Analysis

**Figure 4:**
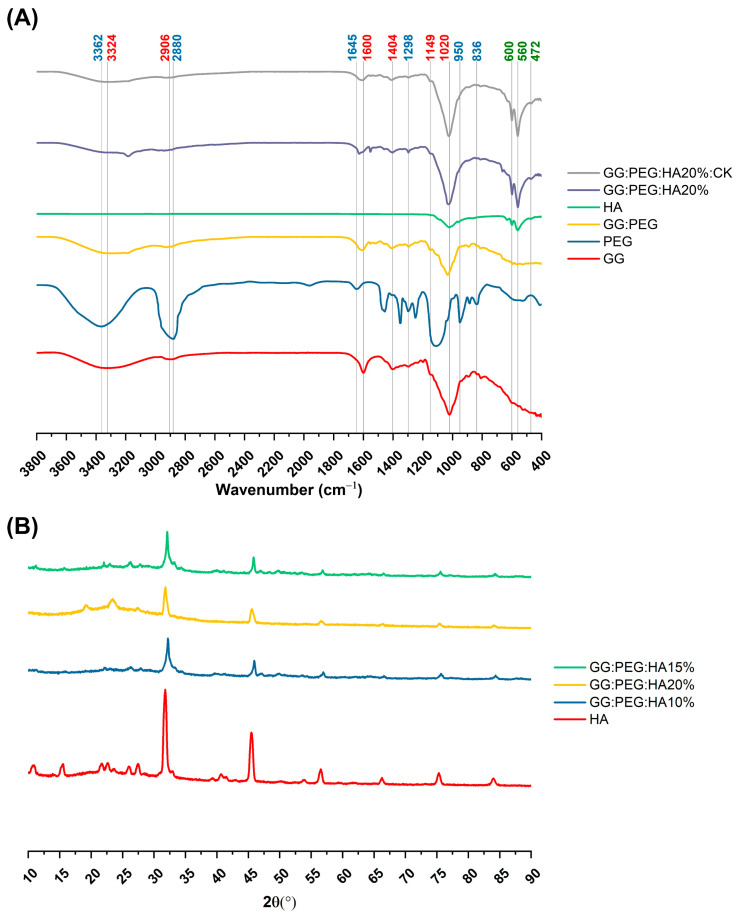
(**A**) FTIR spectra of the prepared scaffolds along with the basic material used (GG, PEG, HA, and CK), the different color of the label indicates the attribution of the specific peak to the material (GG black, PEG red, and HA green). (**B**) XRD spectra to verify the presence of the crystal phase of the HA in the composite scaffolds (HA, CK, control, 20% HA, and 20%HA:CK).

**Figure 5:**
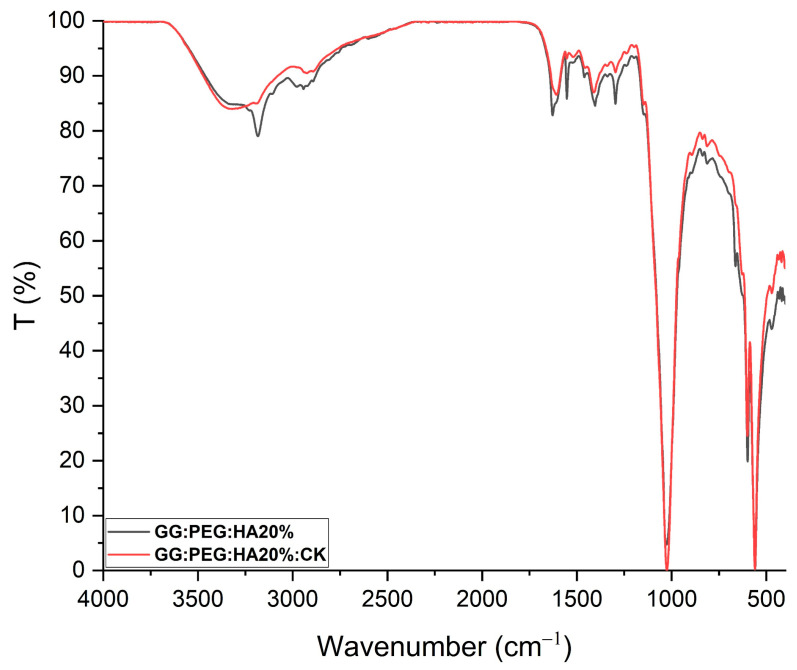
FTIR of GG:PEG:HA20% and GG:PEG:HA20%:CK scaffolds superimposed and normalized. Several peaks well-defined in GG:PEG:HA20% are less defined or not present in case of GG:PEG:HA20%:CK.In particular, a peak located at 3185 cm^−1^ and one located at 1552 cm^−1^ which is less pronounced than the effect of the presence of CK. This may indicate a chemical interaction among CK and the scaffold.

The changes in the chemical composition of the scaffolds were analyzed by FTIR. The GG, HA, GG:PEG, GG:PEG:HA20%, and GG:PEG:HA20%:CK spectra are shown in Figure 4A. The characteristic peaks of GG and PEG were observed in both the bare GG:HA scaffold spectrum and HA-enriched scaffolds (with and without CK). In the GG spectrum, the broad peak at 3324 cm^−1^ corresponds to the –OH group of the glucopyranose ring of GG. The peak centered around 1600 cm^−1^ and 1404 cm^−1^ correspond to the asymmetric and symmetric stretching vibrations of the carboxylate groups (COO-). The alkyl stretching vibrations of the –CH_2_ group’s peak were observed at 2906 cm^−1^. The peaks at 1149 cm^−1^ and 1020 cm^−1^ correspond to the ethereal and hydroxylic C-O stretching vibrations. The bending modes of methyl C-C peaks were observed at 1458 cm^−1^ and 1368 cm^−1^, and the extended peak between 1079 cm^−1^ and 1148 cm^−1^ were attributed to C-O stretching vibrations for alkyl ether [53,54]. In the PEG spectrum, the broad peaks centered around 2880 cm^−1^ and 3362 cm^−1^ were assigned to–CH stretching and –OH stretching, respectively [55]. The presence of both these bands was visible in both the bare GG:PEG scaffold and the composite scaffolds (GG:PEG:HA20% and GG:PEG:HA20%:CK) spectra with some other minor visible changes. In particular, the overlap of the peak centered at 1645 cm^−1^ in the PEG spectrum with the peak at 1600 cm^−1^ in the GG spectrum resulted in a peak centered among these two in all analyzed scaffolds (GG:PEG, GG:PEG:HA20%, and GG:PEG:HA20%:CK). In all scaffolds, we could recognize the peaks at 836 cm^−1^ attributed to the deformation of the –CH group of PEG and the peak individuated at 950 cm^−1^ associated with the vibration of the glucosyl skeleton [56]. In the HA spectra, the peaks at 561 cm^−1^ and 600 cm^−1^ corresponded to the 𝜈_4_ bending mode of PO_4_^3−^ and the anti-symmetric bending motion of the phosphate groups [57]. Both those peaks were clearly recognizable in HA-enriched scaffolds as a clear sign of the HA encapsulation in the GG:PEG matrix. The peak at 472 cm^−1^ associated with the 𝜈_2_ double-degenerated PO_4_^3−^ bending mode [58,59] was also slightly visible in the composite scaffolds (GG:PEG:HA20% and GG:PEG:HA20%:CK). The intense peak was observed in the HA spectrum between 1000 cm^−1^ and 1100 cm^−1^, corresponding to the 𝜈_3_ stretching mode of PO_4_ [60,61,62]. This peak was superimposed to peaks found in GG and PEG in the compound scaffold spectra. The addition of CK resulted in a partial change in the spectrum, as shown in Figure 5. The superposition of the GG:PEG:HA20% and GG:PEG:HA20%:CK spectrum suggests a possible chemical interaction of CK with the scaffold.

The crystallinity of the prepared scaffold was analyzed using XRD, identifying the diffraction angle (2θ) values from the XRD peaks shown in Figure 4B. In particular, XRD was used to determine the presence of HA inside the scaffold without modification to its crystal structure. The semi crystalline structure of the materials is confirmed by the presence of two peaks at 19.16° and 22.56° [63]. The XRD diffractograms of the GG:PEG:HA scaffolds were dominated by the peaks of HA. The diffraction peak at 31.9° was related to the overlapping of {202}, {112}, and {211} planes in the HA crystals. The less intense diffraction peak at 25.9° and 28.1° was assigned to the {002} and {102} plane [64,65]. Typical XRD peaks of HA were distinguished at 46.7° attributed to the {222} plane; 53.1° corresponding to the {401} and {303} planes; and 49.4° assigned to the {231} plane of apatite (JCPDS card no 09-0432). The obtained XRD results clearly showed the successful integration of the HA particles into the composite scaffolds.

### 2.4. Thermal Analysis

**Figure 6:**
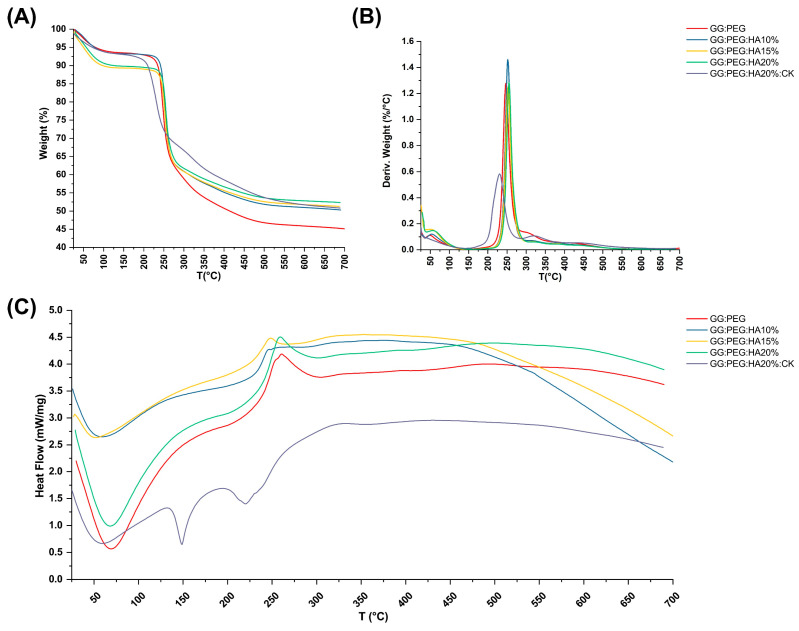
Thermal analysis of the scaffolds. (**A**) TGA and its correspondent (**B**) first derivative. Only some contribution could be clearly distinguished, and the GG:PEG scaffold was the one that lost the highest weight due to the absence of HA. (**C**) DSC analysis—while almost all the samples had the same curve, the sample containing CK was distinguishable for the presence of a second endothermic peak.

The TGA and DSC analysis of scaffolds, performed to investigate their thermal stability, are shown in Figure 6A and Figure 6C, respectively. The TGA of pure PEG and GG were reported in the Supplementary Information (Figure S1). The weight losses are schematically reported in Table 3. It should be noticed that we used the first derivative (Figure 6B) to individuate the change in the slope of the curve and better separate the degradation behavior of the different components of the composite scaffold. The initial weight loss observed in all the samples can be attributed to water evaporation (20–150 °C). The GG:PEG and the sample containing 15% HA particles (GG:PEG:HA15%) exhibited a higher weight loss. This could be probably explained considering a casual variation in the amount of water left in the scaffold in each process (the difference was about 4%). The second weight loss was also present in all the scaffolds, and it was the higher in terms of percentage. This loss was probably related to the degradation of the organic components of the scaffolds and most likely to the crosslinked gellan gum, that, as reported by previous studies, degrade in the 200–300 °C temperature range [59]. Interestingly, the temperature range at which it occurs shifted towards a higher temperature with an increase in the amount of HA particles. We could deduce that HA conferred a higher thermal stability to the scaffolds. It was then possible to recognize a single change in the weight loss slope for the samples with HA without CK (GG:PEG:HA10%, GG:PEG:HA15%, GG:PEG:HA20%), and this loss was assigned to the degradation of the GG:PEG structure because the two contributions were not distinguishable. In the case of absence of HA, an additional change in the slope could be detected (GG:PEG), which can be attributed to the degradation of PEG that is superimposed to the degradation of GG but ends at higher temperatures [60]. The most complex curve is related to CK, in which three variations in the slope were found (and consequently four intervals). It should be noticed that it was not possible to clearly assign the single change in slope to the degradation of single components because they were mixed prior to the crosslinking. So, we indicated it as a degradation of the mixed GG:PEG:CK network in all the cases in which a clear assignment was possible. The last change in the TGA slope was visible only with the presence of CK and thus could be referred to its presence and its interaction with the scaffold. The presence of CK generally decreased the temperature ranges in which the weight loss occurred, so the thermal stability decreased.

The DCS curves are presented in Figure 6C, the initial endothermic peak present in all the scaffold is related to the evaporation of water. In all scaffolds with the exclusion of the one with the addition of CK, an exothermic peak could be detected. The center of this peak moved towards higher temperatures with the increase in the amount of HA added. This exothermic peak suggests the thermal and oxidative decomposition of the scaffold, as suggested by previous works [61,62,63]. In the case in which CK was present, we were able to observe two peaks endothermic peaks typical of native and hydrolyzed GG [61]. Those peaks are both related to decomposition and are not usually detected in case of crosslinked GG [64]. Interestingly, this phenomenon was visible only in the case in which CK was added, a possible hypothesis could be a chemical interaction of CK and the crosslinked GG and a partial hydrolyzation of this last one [61]. This effect was also confirmed by the TGA in which more than one peak was observable in the first derivative, and by the mechanical trial in which there was a slight decrease in the compression Young’s modulus due to the presence of CK.

### 2.5. Swelling and Weight Loss

**Figure 7:**
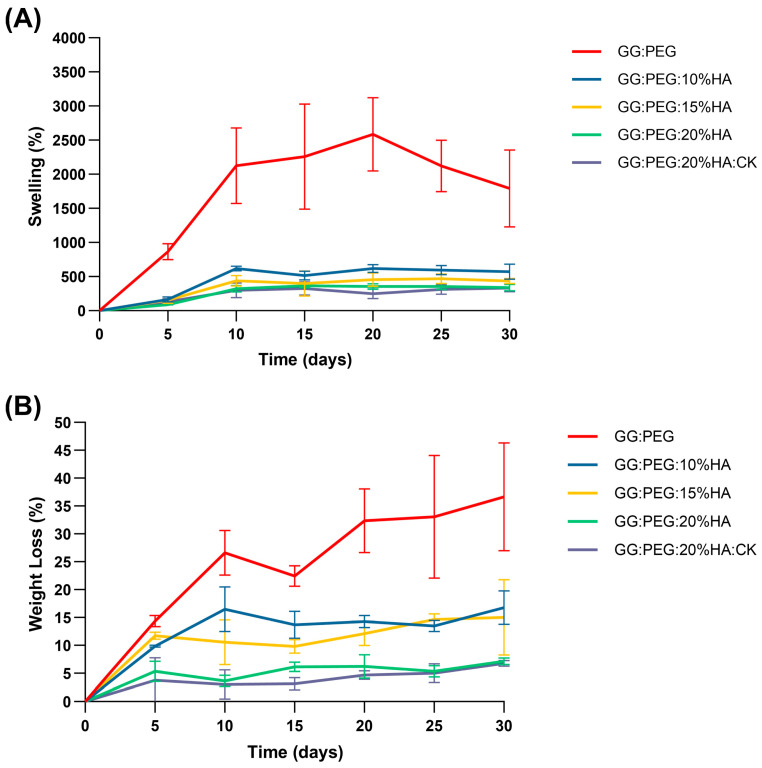
(**A**) Swelling ratio in percentage and (**B**) weight loss. In both cases, an ANOVA test followed by a Turkey comparison has been conducted to test the presence of statistically significant differences.

The percentage swelling and degradation were calculated and are reported in Figure 7A and Figure 7B, respectively. The numerical data are reported in Table 4. The GG:PEG scaffold exhibited a considerably greater swelling ratio compared to all other scaffolds at all time intervals, and decreasing swelling was directly proportional to the amount of added HA. In particular, at day 30, the swelling of GG:PEG was nearly 1800% (so the amount of PBS adsorbed was 18 times the initial weight of the scaffold), while the scaffold with 20% of HA added had a swelling of 340% (3.4 times the initial scaffold weight). This decreasing of the swelling ratio was mainly related to the HA hydrophobicity and is a well-documented phenomenon in the literature [65,66].

The degradation of the scaffolds was also affected by the same factor, which was less significant in relation to the quantity of HA added, as depicted in Figure 7B and Table 4. The same effect was observed in the literature [65,66]. The weight loss data indicate that none of the scaffold completely degraded in the time frame of 30 days. However, the degradation at 30 days for the GG:PEG scaffold was the highest higher (it lost 37% of the initial weight vs. a loss of 7% for the scaffold with 20% of HA).

While a higher swelling ratio ensures a better circulation and may thus be beneficial in terms of the formation of extracellular matrix by cells, it has as a drawback in the decreasing of the mechanical stability as well as the degradation time. In our case, a good compromise resides in the use of the scaffold with the addition of 20% of HA is present regardless of the addition of CK. In fact, CK did not significantly alter the swelling and the degradation behavior of the scaffold over the considered period of 30 days.

### 2.6. Preliminary in Vitro Test: MTT Assay

**Figure 8:**
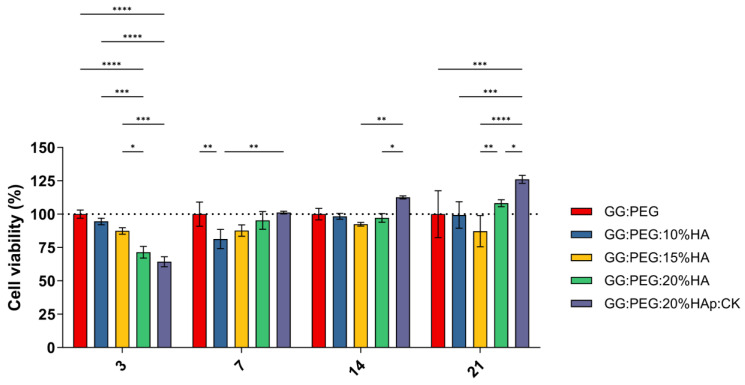
Cell viability determined by MTT assay on day 3, 7, 14, and 21 after seeding. Although all the composite scaffolds initially performed worse than the control, the scaffold with CK eventually outperformed it after 21 days. The significance levels of the ANOVA test were assigned as follows: *p* ≤ 0.05 (*), *p* ≤ 0.01 (**), *p* ≤ 0.001 (***), *p* ≤ 0.0001 (****). A difference was considered statistically significant if *p* ≤ 0.05.

A preliminary in vitro assessment was conducted using the MTT assay on ADSCs cultured on scaffolds and evaluated at 3, 7, 14, and 21 days. The obtained results are shown in Figure 8. The result of the statistical test was reported in Table S1. The GG:PEG scaffold showed significantly higher cell viability after 3 days compared to almost all other scaffolds that included HA. This result was similar to what was already observed in the literature where in other scaffolding systems, in which the HA particles were combined with biomaterials in the first days the scaffold without HA, had better results [67]. This result may be explained by considering the higher swelling ratio on the GG:PEG that allows a higher circulation of nutrients in the first few hours, but also with the direct effect of the presence of HA that partially changes the scaffold surfaces (as shown in Figure 2). At days 7 and 14, the scaffold with the addition of HA showed a response similar to the GG:PEG scaffold with the exclusion of the GG:PEG:HA10% scaffolds that, at day 7, had a cell viability significantly lower than GG:PEG and GG:PEG:HA20%:CK. After 14 days, the scaffold GG:PEG:HA15% resulted in a cell viability lower than GG:PEG:HA20%:CK. At day 21, the samples with the addition of CK showed a significantly higher cell viability than all the other prepared scaffolds, demonstrating that the addition of CK to be beneficial requires at least 21 days. Furthermore, we gathered SEM images at this specific time point (Figure S2), and the trend observed in the MTT assay was also visible in the images. This result well aligns with the limited prior research on the topic, which integrated CK into different scaffolding systems and in which no proved effect of CK on cells was observed in the short term [68,69].

### 2.7. Mineralization

**Figure 9:**
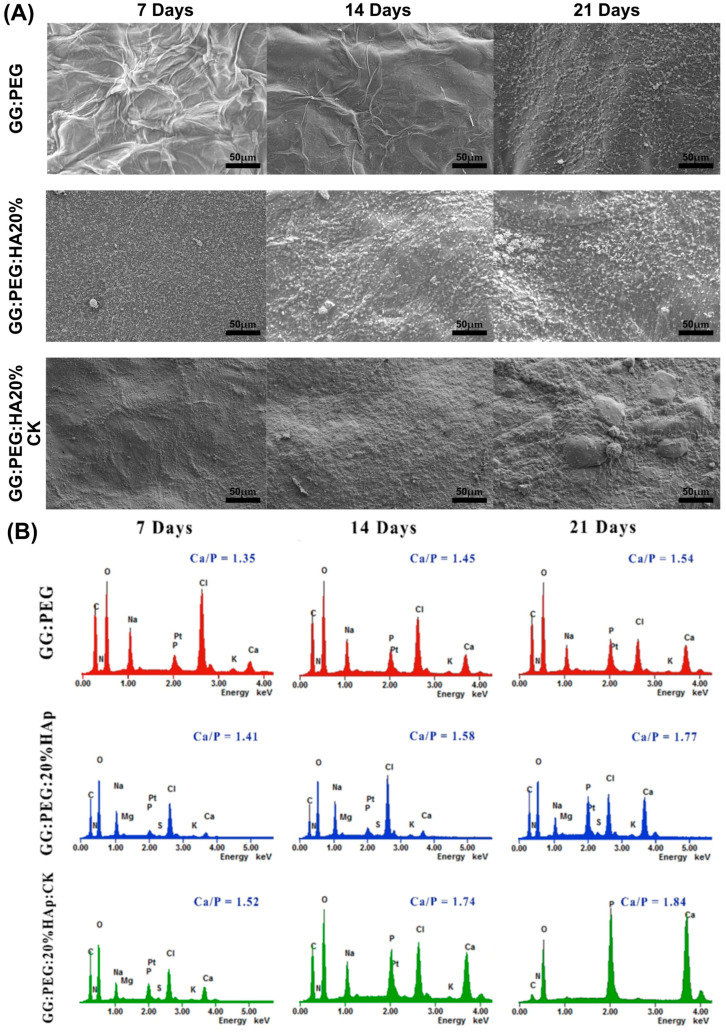
(**A**) SEM images of the surface morphology of the scaffolds and the progressive mineralization occurring over a period of 21 days of immersion in SBF. (**B**) The graph showing the percentage of Ca/P deposition over a period of extended immersion time in the SBF.

The precipitation of calcium and phosphate on the prepared scaffolds were analyzed via incubation in SBF for a period of 21 days. The changes in the surface morphology are shown in the SEM micrographies of Figure 9A. In this case, we tested only the scaffold that gave us the best results in terms of cells viability (GG:PEG:HA20%:CK) versus the correspondent scaffold without CK (GG:PEG:HA20%) and the control (GG:PEG). Both scaffolds with the presence of HA showed a higher mineralization compared to the control in all the tested time points (7, 14, and 21 days). This effect is well known in the literature; in fact, the presence of the HA particles acts as a nucleation center for mineralization. The mineral deposition growth is demonstrated between 7 and 21 days of incubation by the modification in the scaffold morphology. The mineral crystal formation was evenly distributed across the surfaces of the scaffold. The EDX qualitative analysis of Ca-P ratios is reported in Figure 9B. The Ca-P ratio increased with time and moving from the control to the bare scaffold and to the scaffold with the addition of CK. The higher Ca-P ratio was obtained after 21 days for the GG:PEG:HA20%:CK scaffold.

In a previous study, the researchers investigated the ideal ratio between calcium (Ca) and phosphorus (P) [70]. They found that Ca-P ratios ranging from 1.5 to 2 were advantageous for promoting osteoinductivity [70]. However, for osteoconductivity, it was determined that Ca-P ratios between 1 and 1.5 were sufficient [70]. During our study, we discovered that the compositions we tested without CK achieved the optimal range for osteoconductivity at day 7, while only the sample with CK yielded the correct range for osteoinduction. On the other hand, it is worth noting that even the samples without CK were able to achieve the desired osteoinductive range over time. In particular, the GG:PEG:HA20% sample reached the desired range at day 14, while the GG:PEG sample achieved it at day 21.

## 3. Conclusions

A composite bioactive scaffold with potential use in bone regeneration was prepared using gellan gum, polyethylene glycol, hydroxyapatite particles, and the CK derived from ginseng. Different stoichiometric ratios of HA were compared, and their physiochemical and biological properties were fully characterized. From a mechanical standpoint, the addition of both PEG and HA increased the compressive Young’s modulus, whereas the addition of CK slightly decreased it. However, the inclusion of the HA and PEG components resulted in the CK scaffold being mechanically superior to the GG:HA scaffold. The presence of HA also ensured stability against the degradation while it decreased the swelling ratio. The presence of CK was found to improve the viability and proliferation of the cells, as determined by MTT assay, but only after a 14-day incubation period. Two scaffolds, including 20% of HA, were chosen to examine mineralization in SBF (GG:PEG, GG:PEG:20%HAp, and GG:PEG:20%HAp:CK), which showed slightly higher mineralization in the presence of CK. In particular, the Ca-P ratio in the scaffold in the presence of CK yielded the correct range to stimulate osteoinduction since the first tested timepoint. A noticeable difference was present when HA was inserted in the scaffold. In fact, acting as nucleation centers, the HA particles promoted mineralization. This present study represents an initial feasibility study on this new composite scaffold. However, a more in-depth in vitro analysis would be necessary to better understand the material–cell interactions, and an in vivo study will be necessary prior to a clinical translation.

## 4. Materials and Methods

### 4.1. Materials

All the reagents used in this study were of HPLC grade. Gellan gum (GG) (GelzanTM, Sigma-Aldrich Co., St. Louis, MI, USA) with an average molecular weight of 1,000,000 g/mole was purchased. PEG of [Poly (ethylene glycol)] (mw 6000, Sigma-Aldrich) and Trizol (Invitrogen, Life Technologies Co, Groningen, The Netherlands) were used. Hydroxyapatite (HA) nanoparticles were purchased from Sigma Aldrich (St Louis, MO, USA). The main characteristics of this HA powder were characterized by producer (mean diameter of about 70 nm, surface area of 27.6 m^2^/g, purity of 98.9%). Compound K solution was purchased commercially from general bio-Co. Ltd. (Gyeongcheon-myeon, Jeollabuk-do, Republic of Korea) and was completely characterized by the company in a previous research work [71].

All the scaffold components were previously used in tissue engineering and proved to be non-toxic [40,47,72,73].

### 4.2. Scaffolds Preparation

PEG (3 wt%) was dissolved in deionized water (dH_2_O) by continuous stirring at room temperature. Then, GG (1% *w*/*v*) was added to the solution, heated to 90 °C, and stirred at 200 rpm until complete dissolution. The GG:PEG solution was then cooled to 60 °C, and the HA powder was added and gently mixed to obtain the final concentration of 10% *w*/*v*, 15% *w*/*v*, and 20% *w*/*v*. The solution was left to stir for 10 to 15 min at 60 °C to obtain a uniform dispersion of HA. The GG:PEG:HA samples were cooled to 50 °C and poured into petri dish. The samples were then crosslinked by immersing them in CaCl_2_ solution (0.03% *w*/*v*) and placing them in an incubator at 37 °C for 10 min. Cylindrical specimens of hydrogel (8 mm in diameter and 5 mm in height) were obtained using a biopsy punch. The same methodology was used to prepare CK-incorporated scaffolds (PEG:GG:HA:CK). The CK (10 mM) was added at the end, before casting the material into the petri dish. The hydrogels were then kept in PBS for 36 h to remove the excess of salt. Finally, the gels were frozen overnight at −80 °C and freeze-dried for 3 days at −50 °C (Lio5P, *5PASCAL*) to obtain the final scaffold.

### 4.3. Morphological Analysis

Surface morphology of the prepared scaffold was analyzed using scanning electron microscopy (Bio-LV SEM, Hitachi, S-2250N, Tokyo, Japan) after coating with platinum using a plasma sputter (Emscope SC 500 K, Hercules, CA, USA) under argon gas. The pore size distribution was then evaluated by image analysis conducted using ImageJ [74] following a previously developed methodology [18]. The SEM images were analyzed using Fiji (v.1.54i), converting the images to 8-bit and imposing a thresholding and then segmenting the image to obtain the diameter distribution for each scaffold composition as previously described [24]. The mean value within the standard deviation and the median value within the interquartile range were then calculated as statistical measures of the distribution.

### 4.4. Mechanical Test

Three scaffolds for each group were tested both in dry and wet conditions (soaked in water for 6 h at 37 °C). The compressive strength of the scaffolds was measured at room temperature (25 °C) at a speed of 10 mm/min applying a force until the scaffold height was reduced to1 mm using a Universal Testing Machine (TMS-pro, Food Technology Corporation, Sterling, VA, USA). The compressive Young’s modulus was determined as the slope of the initial linear part of the stress–strain curve.

### 4.5. Chemical Analysis

To determine the formation and change in the functional group on the prepared scaffold were analyzed using Fourier-transform infrared spectroscopy using the attenuated total reflectance mode (FTIR-ATR, GX, Perkin Elmer, Waltham, MA, USA) at a wavelength range of 4000–500 cm^−1^ with a 4 cm^−1^ resolution and collecting and mediating 16 scans. Prepared scaffold crystal structure and phase composition were detected using X-ray diffractometer (Bruker D8 Advance DaVinci, Karlsruhe, Germany) equipped with CuKα radiation, produced at 40 kV and 40 mA for phase analysis, crystal size, crystallinity, etc. Data sets were collected in the 2θ range.

### 4.6. Thermal Analysis

Thermogravimetric Analysis (TA Instruments Ltd., Q600, New Castle, DA, USA) was performed for the prepared scaffold. The scaffolds were heated from 30 to 800 °C at a rate of 10 °C/min under nitrogen ambient with a flow rate of 20 mL/min for each group. Primary weight loss of these materials as a function of temperature was recorded using this study. The analysis was conducted by evaluating the derivative of the weight loss to individuate the change in the curve slope and thus distinguish the different components.

Differential scanning calorimetry (Q20, TA instruments, USA) was used to evaluate the thermal behavior of the samples in the range between 30 and 700 °C. The tests were performed under nitrogen flow (50 mL/min) using hermetic lids and a temperature ramp of 10 °C/min.

### 4.7. Swelling and Degradation

Swelling and in vitro degradation studies were carried out for all the prepared scaffolds by immersing them in PBS solution (pH 7.4) and incubating them at 37 °C for a period of 30 days. On days 5, 10, 15, 20, 25, and 30, the swelling ratio and degradation of all the scaffolds were calculated over a period of 30 days, soaked in PBS. The swelling ratio and degradation by the weight loss of all the scaffold composites were calculated by Equations (1) and (2), where *w_i_* is the weight of freeze-dried scaffold composite, *w_s_* is the weight of wet scaffold after swelling at each time point, and wd is the weight of freeze-dried scaffold after swelling at each time point. For each time point, 5 samples were used.

(1) $$Swelling\;\left(\%\right)=\frac{w_{s}-w_{i}}{w_{i}}*100$$

(2) $$Weigth\;loss\;(\%)=\frac{w_{i}-w_{d}}{w_{i}}*100$$

### 4.8. Preliminary In Vitro Test: MTT Assay and SEM Imaging

To investigate the effect of the sonication of the dbPTs on cell proliferation, commercial adipose-derived stem cells (ADSCs) were employed (Lonza, Basel, Switzerland). Upon thawing, cells were expanded in α-MEM 15% FBS at 37 °C, 5% CO_2_, and 95% O_2_, and the medium was changed every three days. At the start of the experiment, cells were harvested with trypsin/EDTA solution 0.25% (Biochrom, Berlin, Germany) and seeded onto the composite scaffold at a density of 1 × 10^5^ (cells/scaffold), allowing their attachment for about 1 h in the incubator. After that, the medium was added until scaffold coverage and then incubated. The GG:PEG scaffold was used as positive control for the culture. This allowed us to directly verify the impact of adding other materials on cells. 3-[4,5-dimethylthiazol-2-yl]-2,5-diphenyltetrazoliumbromide (MTT) assay was performed after 1, 4, 7, 14, and 21 days. The MTT solution (sigma, 50 mg/L in PBS) was added, followed by incubation at 37 °C, at 5% CO_2_ for 4 h. The media was removed and replaced with 1 mL dimethyl sulfoxide (DMSO, Sigma-Aldrich, St. Louis, MO, USA) to dissolve formazan crystals. Then, they were measured using microplate reader (E-max, Molecular device, San Jose, CA, USA) at 570 nm to detect the absorbance of samples.

SEM images were taken on treated samples. Briefly, at the specific time point, the cells’ treated scaffolds were rinsed with PBS and fixed using a 2.5% solution of glutaraldehyde (Sigma-Aldrich, USA) overnight. Scaffolds were then dehydrated soaking them in an increasingly high percentage of ethanol solution (30, 50, 70, 80, 90, 100% aqueous ethanol) for 30 min at each step. Finally, the samples were lyophilized after freezing overnight at −80 °C. The dried samples were coated with platinum under argon gas using sputter (Emscope, SC 500 K, Hercules, CA, USA) and visualized using SEM (Bio-LV SEM, Hitachi, S-2250N, Tokyo, Japan).

### 4.9. Mineralization

The prepared scaffolds were immersed in simulated body fluid (SBF) according to Kokubo’s method [48]. The SBF molar concentration was intended to mimic human blood plasma. The pH of the SBF was corrected to 7.4 and were maintained at 36 °C, SBF were prepared using Na^+^ 142.0 mM, Ca^2+^ 2.5 mM, Mg^2+^ 1.5 mM, K^+^ 5.0 mM, Cl^−^ 147.8 mM, HPO_4_^2−^ 1.0 mM, HCO_3_^−^ 4.2 mM, SO_4_^2−^ 0.5 mM. The scaffolds were placed in a polystyrene 8 well plate and filled with SBF until the scaffold was immersed in it and incubated at 36 °C for 30 days. At each time point, the scaffolds were retrieved and rinsed in distilled water and frozen at −80 °C prior to lyophilization. To evaluate the surface morphology and verify the mineralization, a secondary electron microscope with energy dispersive X-ray spectroscopy (SEM-EDX, Bio-LV SEM, Hitachi, S-2250N, Tokyo, Japan) was used.

## Figures and Tables

**Table 1:** Descriptive statistic of the pore diameter distribution. The mean within the standard deviation, the minimum and the maximum value, and the median within the first (Q1) and third (Q3) quartile showing the limits of the interquartile range in which 50% of the collected diameter resides. The other two statistics have been used to compare the shape of the distribution, and the skewness showing if the distribution is symmetrical towards the mean and the kurtosis, showing how “heavy” is the tail of the distribution.

Composition	Mean µm	St. Dev. µm	Skew	Kurt	Min µm	Q1 µm	Median µm	Q3 µm	Max µm
GG:PEG	197.2	102.1	0.79	0.73	25.7	118.7	188.3	260.0	557.7
GG:PEG:HA10%	180.4	88.4	0.47	−0.69	46.2	106.7	169.9	254.9	410.5
GG:PEG:HA15%	196.2	90.2	0.61	0.23	49.6	133.4	190.5	238.4	463.2
GG:PEG:HA20%	152.3	71.4	1.16	1.52	32.9	102.6	137.3	176.7	399.1
GG:PEG:HA20%:CK	144.8	116.4	2.63	9.70	23.0	68.9	112.1	182.6	728.5

**Table 2:** Values of the mean compressive Young’s modulus and its standard deviation. Percentage loss passing from the dry to the wet condition.

	GG	GG:PEG	GG:PEG:HA10%	GG:PEG:HA15%	GG:PEG:HA20%	GG:PEG:HA20%:CK
E_Dry_ (kPa)	408 ± 16	959 ± 34	1078 ± 46	1084 ± 20	1494 ± 73	1591 ± 33
E_Wet_ (KPa)	86 ± 3	438 ± 27	872 ± 18	1018 ± 45	1152 ± 54	860 ± 36
Loss (%)	78	54	19	6	23	46

**Table 3:** Weight loss and correspondent temperature range revealed by thermogravimetric analysis and their derivatives. In the sample without CK, the range shifted towards higher temperature with the increase in the amount of HA.

Scaffold	Temperature Range (°C)	Weight Loss (%)	Assignment
GG:PEG	25–125	6.3	Water Loss
	125–175	0.36	-
	175–283	31.1	Deg. GG
	283–345	8.1	Deg. PEG
	345–700	9.04 (Residue 45.1)	Deg. GG:PEG
GG:PEG:HA10%	25–125	6.6	Water Loss
	125–200	0.33	-
	200–287	31.1	Deg. GG
	287–700	11.62 (Residue 50.3)	Deg. GG:PEG
GG:PEG:HA15%	25–130	10.62	Water Loss
	130–200	0.36	-
	200–292	27.74	Deg. GG
	292–700	10.1 (Residue 51.3)	Deg. GG:PEG
GG:PEG:HA20%	24–140	10.13	Water Loss
	140–208	0.45	-
	208–303	27.88	Deg. GG
	303–700	9.18 (Residue 52.36)	Deg. GG:PEG
GG:PEG:HA20%:CK	25–110	6.41	Water Loss
	110–150	0.57	-
	150–284	24.95	Deg. GG
	284–385	8.66	Deg. PEG
	385–529	6.46	Deg. GG:PEG:CK
	529–700	2.13 (Residue 50.82)	Deg. GG:PEG:CK

**Table 4:** Swelling ratio and degradation in percentage over a time of 30 days. The values are reported as mean and standard deviation over calculated over five samples for each condition.

Sample	5 Days	10 Days	15 Days	20 Days	25 Days	30 Days
**Swelling (%)**						
GG:PEG	865 ± 118	2124 ± 553	2259 ± 769	2584 ± 537	2123 ± 378	1792 ± 563
GG:PEG:HA10%	164 ± 39	619 ± 34	516 ± 66	620 ± 58	595 ± 68	572 ± 109
GG:PEG:HA15%	157 ± 47	439 ± 79	399 ± 184	456 ± 96	470 ± 73	435 ± 36
GG:PEG:HA20%	87 ± 4	323 ± 49	364 ± 29	356 ± 40	356 ± 43	338 ± 46
GG:PEG:HA20%:CK	131 ± 44	299 ± 107	324 ± 97	249 ± 70	312 ± 70	333 ± 56
**Degradation (%)**						
GG:PEG	14 ± 1	27 ± 4	22 ± 2	32 ± 6	33 ± 11	37 ± 10
GG:PEG:HA10%	10 ± 1	16 ± 4	14 ± 2	14 ± 1	13 ± 1	17 ± 3
GG:PEG:HA15%	12 ± 1	11 ± 4	10 ± 1	12 ± 2	15 ± 1	15 ± 7
GG:PEG:HA20%	5 ± 2	4 ± 1	6 ± 1	6 ± 2	5 ± 1	7 ± 1
GG:PEG:HA20%:CK	4 ± 4	3 ± 3	3 ± 1	5 ± 1	5 ± 2	7 ± 1

## Data Availability

Data are available from the authors upon reasonable request.

## Supplementary Materials

The following supporting information can be downloaded at: https://www.mdpi.com/article/10.3390/gels10040257/s1, Figure S1: TGA of the raw PEG 6000 and GG; Figure S2: SEM on scaffolds with fixed cells at day 21; Table S1: Result of the post-hoc Tukey’s multiple comparisons test on MTT assay results.
