# Supplementary material for: A Gellan Gum, Polyethylene Glycol, Hydroxyapatite Composite Scaffold with the Addition of Ginseng Derived Compound K with Possible Applications in Bone Regeneration
Source: Gels. 2024 Apr 10;10(4):257. doi: 10.3390/gels10040257 (PMC11049517; doi:10.3390/gels10040257)
Supplement: Supplementary file 1 [file gels-10-00257-s001.zip › gels-2931132-supplementary.pdf]

Supporting Information

A Gellan Gum, Polyethylene Glycol, Hydroxyapatite Composite Scaffold with the Addition of Ginseng Derived Compound K with Possible Applications in Bone Regeneration

Muthukumar Thangavelu [1,*,†], Pil-Yun Kim [2,†], Hunhwi Cho [2], Jeong-Eun Song [2], Sunjae Park [3], Alessio Bucciarelli [4,*] and Gilson Khang [2,*]

1 Linkocare Life Sciences AB, Mjärdevi Science Park, 583 30 Linköping, Sweden
2 Department of Bionanotechnology and Bio-Convergence Engineering, Jeonbuk National University, 567 Baekje-daero, Deokjin-gu, Jeonju-si 54896, Jeonbuk, Republic of Korea
3 Department of Polymer Nano Science & Technology and Polymer Materials Fusion Research Center, Jeonbuk National University, 567 Baekje-daero, Deokjin-gu, Jeonju-si 54896, Jeonbuk, Republic of Korea
4 Laboratorio RAMSES, IRCCS Istituto Ortopedico Rizzoli, Via di Barbiano 1/10, 40136 Bologna, Italy
* Correspondence: auromuthu@gmail.com (M.T.); alessio.bucciarelli@ior.it (A.B.); gskhang@jbnu.ac.kr (G.K.)
† These authors contributed equally to this work.

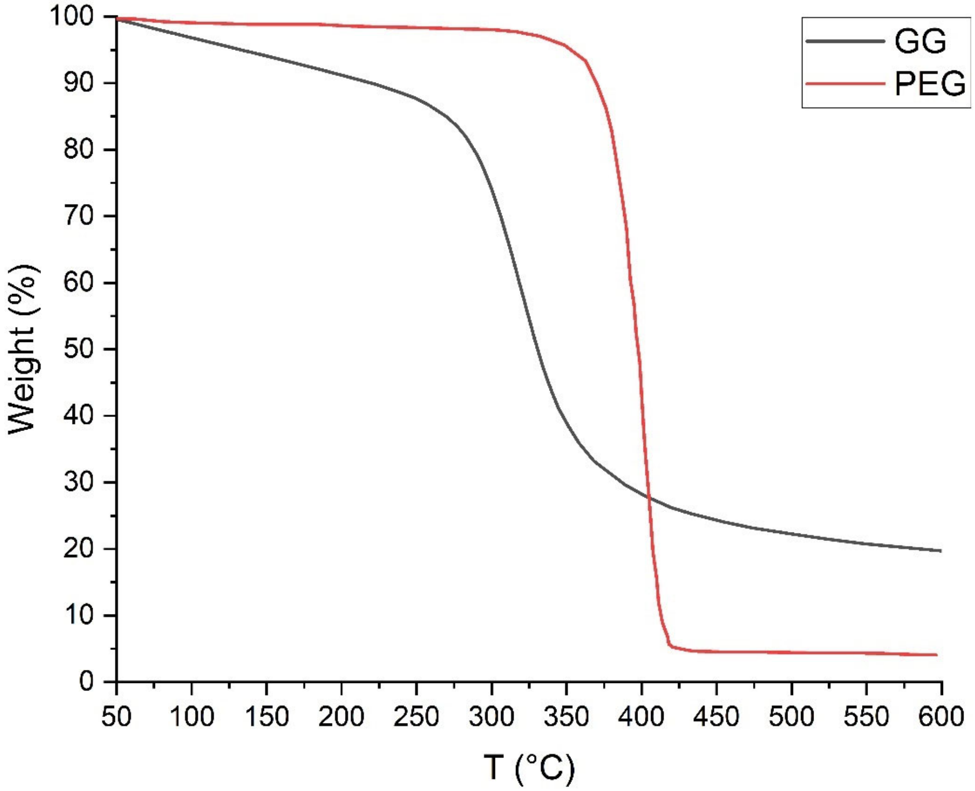

Figure S1: TGA of the raw PEG 6000 and GG. In the composite scaffold this results in a curve with a variable slope as effectively observed in GG:PEG scaffold.

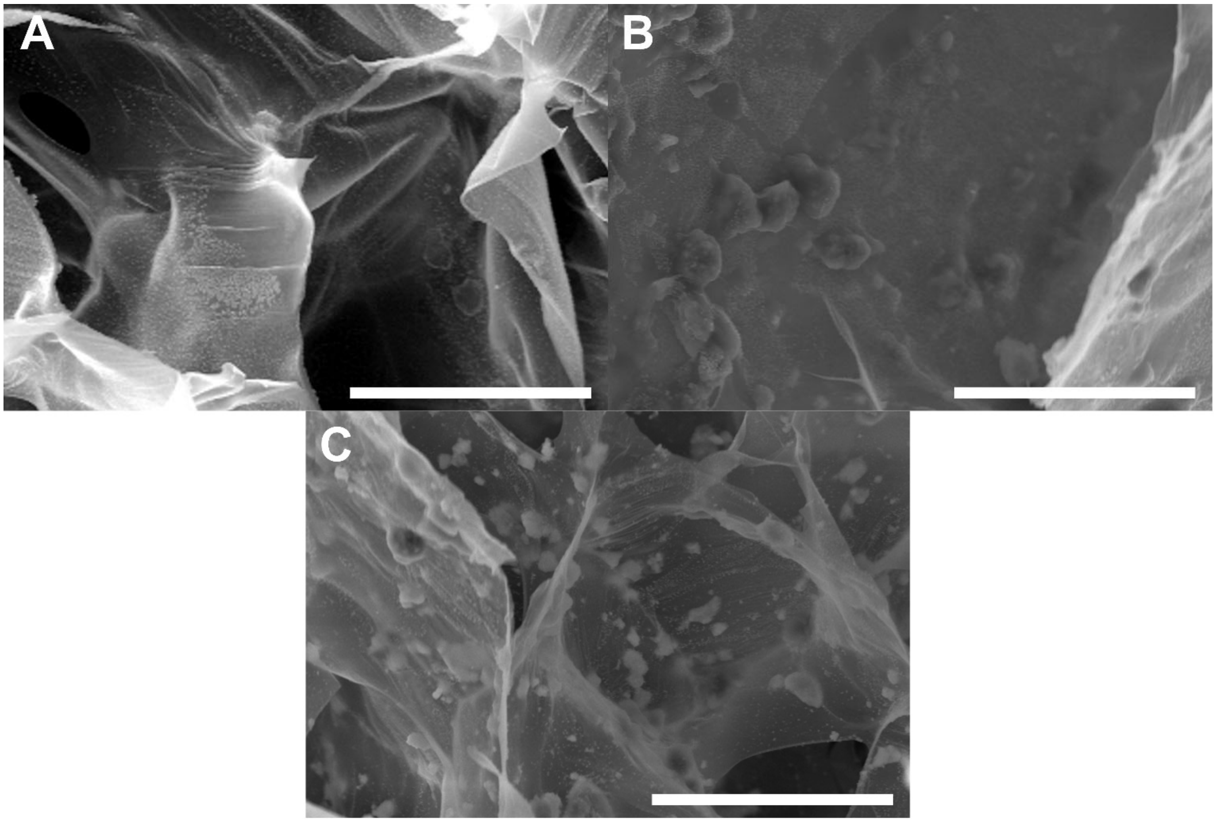

Figure S2: SEM on scaffolds with fixed cells at day 21. We compared the (**A**) GG:PEG, (**B**) GG:PEG:HA20% and (**C**) GG:PEG:HA20%:CK. The images confirmed the same trend reported by the MTT assay an increasing number of cells were populating the last scaffold.

Table S1: Result of the post-hoc Tukey's multiple comparisons test on MTT assay results.

Tukey's multiple comparisons test	Mean Diff.	95.00% CI of diff.	Summary	Adjusted P Value
**Day 1**				
GG:PEG vs. GG:PEG:HA10%	9.505	-7.226 to 26.24	ns	0.4996
GG:PEG vs. GG:PEG:HA15%	-13.28	-30.01 to 3.455	ns	0.1802
GG:PEG vs. GG:PEG:HA20%	24.82	8.093 to 41.55	**	0.001
GG:PEG vs. GG:PEG:HA20%:CK	29.84	13.11 to 46.57	****	<0.0001
GG:PEGHA10% vs. GG:PEG:HA15%	-22.78	-39.51 to -6.050	**	0.0029
GG:PEG:HA10% vs. GG:PEG:HA20%	15.32	-1.413 to 32.05	ns	0.0875
GG:PEG:HA10% vs. GG:PEG:HA20%:CK	20.33	3.604 to 37.06	**	0.01
GG:PEG:HA15% vs. GG:PEG:HA20%	38.1	21.37 to 54.83	****	<0.0001
GG:PEG:HA15% vs. GG:PEG:HA20%:CK	43.12	26.38 to 59.85	****	<0.0001
GG:PEG:HA20% vs. GG:PEG:HA20%:CK	5.016	-11.71 to 21.75	ns	0.914
**Day 3**				
GG:PEG vs. GG:PEG:HA10%	5.498	-11.23 to 22.23	ns	0.8839
GG:PEG vs. GG:PEG:HA15%	12.61	-4.117 to 29.34	ns	0.2224
GG:PEG vs. GG:PEG:HA20%	28.55	11.82 to 45.28	***	0.0001
GG:PEG vs. GG:PEG:HA20%:CK	35.67	18.94 to 52.40	****	<0.0001
GG:PEGHA10% vs. GG:PEG:HA15%	7.116	-9.615 to 23.85	ns	0.7493
GG:PEG:HA10% vs. GG:PEG:HA20%	23.06	6.326 to 39.79	**	0.0026
GG:PEG:HA10% vs. GG:PEG:HA20%:CK	30.17	13.44 to 46.90	****	<0.0001
GG:PEG:HA15% vs. GG:PEG:HA20%	15.94	-0.7906 to 32.67	ns	0.0688
GG:PEG:HA15% vs. GG:PEG:HA20%:CK	23.05	6.323 to 39.78	**	0.0026
GG:PEG:HA20% vs. GG:PEG:HA20%:CK	7.113	-9.617 to 23.84	ns	0.7495
**Day 7**				
GG:PEG vs. GG:PEG:HA10%	18.65	1.920 to 35.38	*	0.0218
GG:PEG vs. GG:PEG:HA15%	12.35	-4.380 to 29.08	ns	0.2409
GG:PEG vs. GG:PEG:HA20%	4.682	-12.05 to 21.41	ns	0.9318
GG:PEG vs. GG:PEG:HA20%:CK	-1.215	-17.95 to 15.52	ns	0.9996
GG:PEGHA10% vs. GG:PEG:HA15%	-6.3	-23.03 to 10.43	ns	0.8231
GG:PEG:HA10% vs. GG:PEG:HA20%	-13.97	-30.70 to 2.762	ns	0.1427
GG:PEG:HA10% vs. GG:PEG:HA20%:CK	-19.87	-36.60 to -3.135	*	0.0125
GG:PEG:HA15% vs. GG:PEG:HA20%	-7.669	-24.40 to 9.061	ns	0.6941
GG:PEG:HA15% vs. GG:PEG:HA20%:CK	-13.57	-30.30 to 3.164	ns	0.1637
GG:PEG:HA20% vs. GG:PEG:HA20%:CK	-5.897	-22.63 to 10.83	ns	0.8553
**Day 14**				
GG:PEG vs. GG:PEG:HA10%	1.64	-15.09 to 18.37	ns	0.9987
GG:PEG vs. GG:PEG:HA15%	7.577	-9.154 to 24.31	ns	0.7036
GG:PEG vs. GG:PEG:HA20%	2.827	-13.90 to 19.56	ns	0.989
GG:PEG vs. GG:PEG:HA20%:CK	-12.59	-29.32 to 4.142	ns	0.2241
GG:PEGHA10% vs. GG:PEG:HA15%	5.937	-10.79 to 22.67	ns	0.8522
GG:PEG:HA10% vs. GG:PEG:HA20%	1.187	-15.54 to 17.92	ns	0.9996
GG:PEG:HA10% vs. GG:PEG:HA20%:CK	-14.23	-30.96 to 2.502	ns	0.1304

GG:PEG:HA15% vs. GG:PEG:HA20%	-4.75	-21.48 to 11.98	ns	0.9284
GG:PEG:HA15% vs. GG:PEG:HA20%:CK	-20.17	-36.90 to -3.435	*	0.0108
GG:PEG:HA20% vs. GG:PEG:HA20%:CK	-15.42	-32.15 to 1.315	ns	0.0843
**Day 21**				
GG:PEG vs. GG:PEG:HA10%	0.6535	-16.08 to 17.38	ns	>0.9999
GG:PEG vs. GG:PEG:HA15%	12.75	-3.976 to 29.49	ns	0.2129
GG:PEG vs. GG:PEG:HA20%	-8.177	-24.91 to 8.553	ns	0.6411
GG:PEG vs. GG:PEG:HA20%:CK	-26.06	-42.79 to -9.329	***	0.0005
GG:PEGHA10% vs. GG:PEG:HA15%	12.1	-4.630 to 28.83	ns	0.2594
GG:PEG:HA10% vs. GG:PEG:HA20%	-8.831	-25.56 to 7.900	ns	0.5714
GG:PEG:HA10% vs. GG:PEG:HA20%:CK	-26.71	-43.44 to -9.983	***	0.0004
GG:PEG:HA15% vs. GG:PEG:HA20%	-20.93	-37.66 to -4.201	**	0.0075
GG:PEG:HA15% vs. GG:PEG:HA20%:CK	-38.81	-55.55 to -22.08	****	<0.0001
GG:PEG:HA20% vs. GG:PEG:HA20%:CK	-17.88	-34.61 to -1.152	*	0.0307
